# Effects of vegetation restoration on distribution characteristics of heavy metals in soil in Karst plateau area of Guizhou

**DOI:** 10.7717/peerj.15044

**Published:** 2023-03-17

**Authors:** Yunjie Wu, Xin Tian, Runze Wang, Mingyi Zhang, Shuo Wang

**Affiliations:** 1 College of Eco-Environmental Engineering, The Institute of Karst Wetland Ecology, Guizhou Minzu University, Guiyang, Asia, China; 2 Department of Mechanical and Electrical and Urban Construction, Guizhou Vocational College of Agriculture, Qingzhen, Asia, China

**Keywords:** Karst, Caohai, Vegetation restoration, Heavy metals

## Abstract

In southwest China, vegetation restoration is widely used in karst rocky desertification control projects. This technology can effectively fix the easily lost soil, gradually restore the plant community and improve soil fertility. However, the change law of soil heavy metals in the restoration process remains to be further studied. Therefore, in this work, Guizhou Caohai Nature Reserve as a typical karst area was taken as the research object to investigate the influence of vegetation restoration technology on repairing soil heavy metal pollution. The spatial distribution characteristics of soil heavy metals (chromium, nickel, arsenic, zinc, lead) before and after vegetation restoration in karst area were studied by comparative analysis and linear stepwise regression analysis. The main influencing factors and spatial distribution characteristics of heavy metals in karst area were further discussed. The results showed that: (1) heavy metals in karst soils are affected by surface vegetation, root exudates, microorganisms and leaching. Only heavy metals nickel (Ni) and lead (Pb) showed the tendency of surface enrichment and bottom precipitation enrichment in non-karst soils. Path analysis suggested that non-metallic soil factors such as soil bulk density (BD), total nitrogen (TN) and ammonium nitrogen (NH_4_^+^-N) had direct effect on the content of heavy metals in soil. (2) The proportion of 0.25–2 mm aggregates in the surface soil of vegetation restoration belt was more than 40%, and the proportion of surface soil ≤2 mm aggregates in this increased to 83% and 88%, respectively, which could improve the soil structure and properties effectively. (3) Vegetation restoration effectively restored the nutrient elements such as carbon and nitrogen in the soil, and enhanced the soil material circulation. Furthermore the content of heavy metals in the surface soil higher than that in the 10–20 cm soil layer. Plant absorption, biosorption mechanism of microorganisms, coupling of root exudates, dissolution of soil soluble organic carbon and pH make the contents of heavy metals Cr, Ni and Pb in vegetation restoration belt slightly lower than those in karst soil. At the same time, affected by vegetation coverage, residual heavy metals in soil are further leached by surface runoff. Therefore, the content of heavy metals in soil could reduce combined heavy metal enrichment plants for extraction with remediation. This study elucidates the advantages and remedy mechanism of vegetation restoration in the remediation of heavy metal contaminated soils in Caohai area of Guizhou, and this plant activation and enrichment extraction remediation technology would be popularized and applied in the remediation of heavy metal contaminated soils in other karst areas.

## Introduction

Heavy metals are regarded as the main harmful trace elements because of their high toxicity, long residence time and long-lasting bioavailability. Soil is an important carrier of heavy metals, causing heavy metals to accumulate in soil. which can be absorbed by plants into the food chain or migrated into water and atmosphere. When excessive heavy metals enter the soil layer, it can lead to the decrease of soil productivity and soil quality (Su et al., 2022; Wei, Wang & Daniel, 2016). At the same time, the soil seriously polluted by heavy metals may become a long-term pollution source of groundwater and ecosystem (Konyshev et al., 2020; Li et al., 2009). Therefore, heavy metal pollution in soil is becoming an increasingly serious environmental problem (Zhong et al., 2018; Yang, Wu & Yang, 2022; Zhang et al., 2022).

Recently, a great deal of research have studied on the sources and contents, effects on crops and spatial distribution of heavy metals in different kinds of soil, such as, urban soil (Han & Xu, 2022), farmland soil (Zhang et al., 2014, 2018), industrial park soil (Yang et al., 2018; Zhang et al., 2020a) and farmland around mining area (Zhou et al., 2022). These results shown that heavy metal activity is closely related to soil properties. Among various properties, pH value is an important factor affecting the bioavailability of soil bound heavy metals (Zhang et al., 2021). To solve the major practical problems including hostile environment and hindered agricultural production caused by soil heavy metal pollution, a lot of studies focused on the migration and enrichment of heavy metal pollutants in soil (Yao et al., 2021; Li, Jin & Gu, 2011; Wang et al., 2013) as well as the treatment of heavy metal contaminated soil with phytoremediation technology (Zhou et al., 2022; He et al., 2020).

Among them, the status of soil heavy metals pollution in karst areas in different land and vegetation cover types appeared in karst areas (Han et al., 2022; Liu et al., 2022; Sun et al., 2020). Such as natural background, agricultural activity area (Zhang et al., 2021; Han et al., 2012; Zeng et al., 2019), mining area (Zhou et al., 2022; Zhang et al., 2022; Wang et al., 2022b; Qi et al., 2022) has become a major thrust of research. The research content is mainly based on the distribution characteristics of soil heavy metal content or how to reduce the heavy metal content in soil. It is considered that the exploitation of mineral resources, excessive application of pesticides and fertilizers and other human activities are the main sources of heavy metal pollution in karst areas.

Very recently, complex geology and geomorphology, variability of soil physical and chemical properties, migration and transformation process of pollutants, incomplete remediation of existing soil pollution have been considered as the mainly limiting factors of soil pollution remediation (Guo et al., 2022). At present, the research on soil heavy metal pollution in karst areas of southwest China is mainly focused on two aspects: one is the changes of soil structure (aggregates), soil organic matter and other nutrients (Xu et al., 2017; Sun et al., 2009) under the influence of heavy metal pollution. The second is the distribution characteristics of heavy metals in soil (Zhang et al., 2021, 2022) under different land use types such as farmland and mining area (Zhu, Xiao & Guo, 2018), removal effect (Zhou et al., 2022) and remediation technology exploration (Li, 2020). However, research on the mechanism of phytoremediation technology for heavy metals in soil is limited. Especially, the application of phytoremediation technology in karst areas is lacked due to the complexity of geology and geomorphology in karst areas. Hence, it is necessary to explore the distribution characteristics, migration and transformation mechanism of heavy metals in karst soil by combining factors such as vegetation restoration, soil nutrients and structure.

Hence, the Caohai area of Guizhou Province as a typical karst area was taken as the research target region, and the aims of the present work are (1) to investigate the spatial distribution characteristics of soil heavy metals (chromium, nickel, arsenic, zinc, lead), which might be affected by vegetation restoration and the properties of karst area; (2) to explore the main factors such as soil physical and chemical properties, microbial activity and plant adsorption affecting heavy metals migration and mechanisms on the basis of distribution characteristics analysis. This study would provide support for ecological environment protection and land management in karst area.

## Materials and Methods

### Regional profile of study

**Figure 1:**
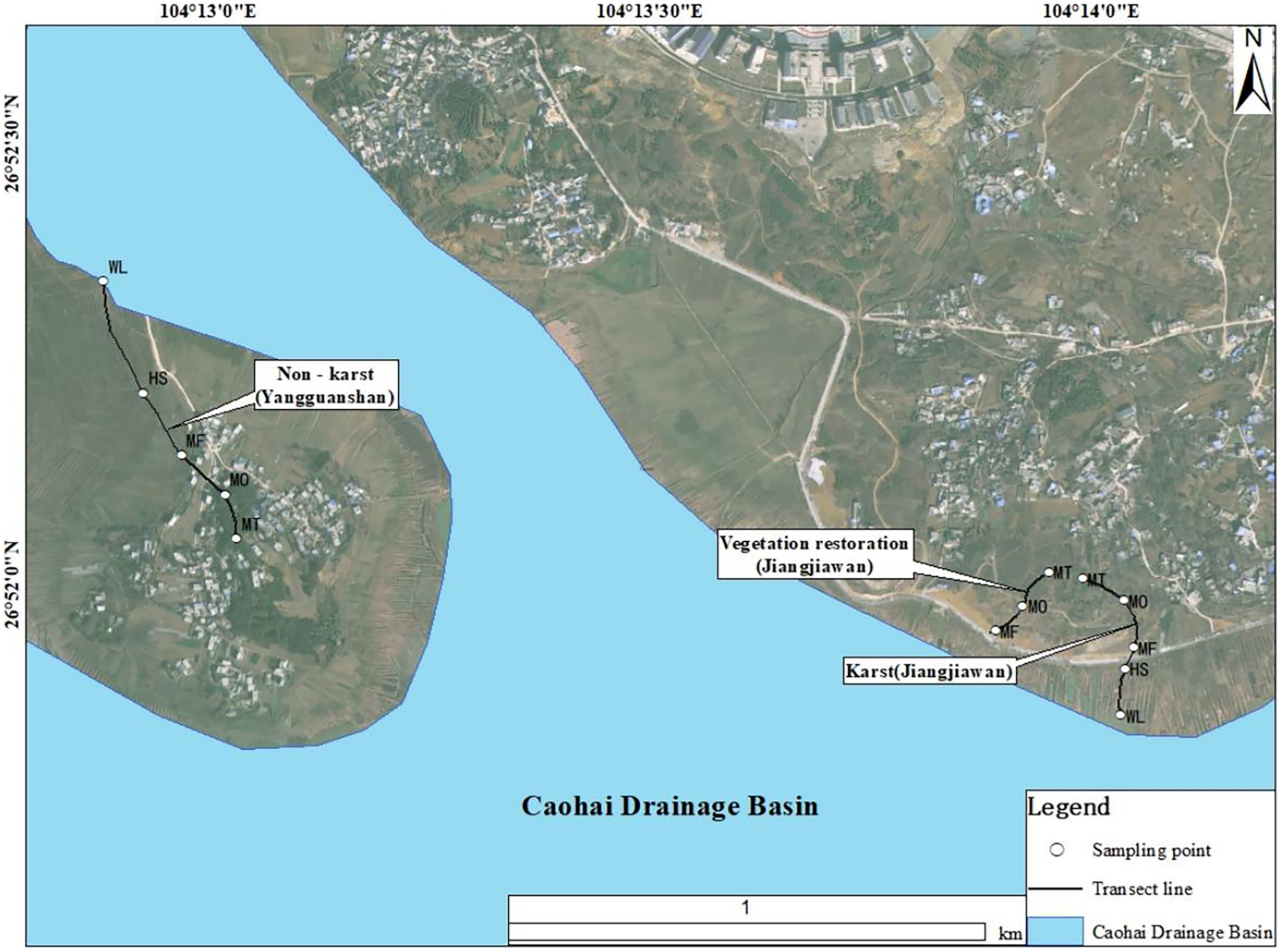
Location of the study area.

The study area is located in the Caohai National Nature Reserve in Weining, Guizhou (26°49: 26°53 N, 104°12: 104°18°E) (Fig. 1). The total area of the Caohai National Nature Reserve is about 120 km^2^, the average elevation is about 2,171.7 m, and the drop between the lake and the surrounding mountains is 100–120 m. It belongs to the subtropical monsoon area with an annual temperature of 10.6 °C and an average annual rainfall of about 1,000 mm. The soil types are mainly yellow soil and limestone soil, and the rock exposure rate is more than 75%. It has well-developed soil surface, stone pits and other niches, which is a typical karst area. The unique geological structure and soil-forming materials of carbonate rocks in karst area are lacking, resulting in shallow and discontinuous soil layer, and soil erosion leads to soil degradation easily (He et al., 2019).

In the past two decades, the two-way evolution of land use types from nature to cultivated land has seriously led to rocky desertification, further increase of soil erosion, aggravation of soil physical clay change and decrease of vegetation coverage and so on. Among them, the sources of heavy metals in the Caohai area are mainly geological weathering and soil forming process and man-made factors (Yang, Xin & Tian, 2022; Xu et al., 2015). Local measures such as “vegetation restoration, returning farmland to forest and grassland” have been taken to control rocky desertification areas in the Caohai area and good progress has been made. In 2016, the “Vegetation Restoration Project in the Rain gathering area on the North Bank of Caohai” was implemented. The project aims at the ecological restoration project of serious rocky desertification, low vegetation coverage and serious soil erosion on the north slope of Caohai.

### Sample collection

This study is based on the “Mian Shan vegetation Restoration Project on the North Bank of Caohai”. According to the “National soil Environmental quality Construction Land soil pollution risk Control Standard (trial) (GB36600-2018)” and “National soil Environmental quality Agricultural Land soil pollution risk Control Standard (trial) (GB 15618-2018)”, and combined with the current situation of field investigation of soil heavy metals in Caohai area. Finally, five kinds of heavy metals including chromium, nickel, arsenic, zinc and lead (Cr, Ni, As, Zn and Pb) were selected for quantitative analysis. It was noted that the contents of cadmium (Cd) and mercury (Hg) in this study area too low to detect, and their distribution was not analyzed. Three geomorphological belts including one non-karst belt (Yangguan Mountain in Caohai region), one karst rocky desertification zone (Jiangjiawan) and one vegetation restoration belt (Jiangjiawan) were selected. The two sample zones in karst area have the same habitat, and half of area has vegetation restoration and half without vegetation restoration for comparative study. Karst and vegetation restoration areas are connected by wetland, therefore they owing the same data of the shore and the wetland. Before restoration (karst), the vegetation communities were mainly dwarf shrubs and herbs, and the distribution was relatively uniform. The shrub communities including *Cotoneasterhissaricus and Rberiswilsona*, and the herb layer communities were mainly *Hemisiacarvifol* and *Perusdulouxi* (Wu et al., 2013; Zhang et al., 2013). In particular, the duration time of vegetation restoration transect in vegetation restoration belt is 3–4 years, and mainly artificial vegetation (Yunnan poplar and herbs). The geographical location of the transect and the present situation of vegetation are shown in Table 1. The soil type in Jiangjiawan area is lime soil.

**Table 1:** Location and vegetation status of the transect.

Transect	Latitude longitude	Altitude/m	Vegetation coverage/%	Soil type	Simpson diversity index	Phytocoenosium
Non-karst (NK)	104°12′1.82″–104°13′1.82″E 26°52′3.10″–26°52′20.60″N	2,174–2,196 m	80–85%	Yellow soil	0.75	Arbor community: dominated by Yunnan pine (*Pinus yunnanensis*), associated with cypress (Sabina chinensis), Chinese fir (Cunninghamia lanceolate), *etc*. Shrub community: mainly juniper (*Juniperus rigida*) and firethorn (*Pyracantha fortuneana*); herbaceous plants: *Eleusine indica*, *Oenanthe javanica* and Juncus effuses).
Karst (K)	104°14′0.33″–104°14′2.81″E 26°51′51.11″–26°52′0.38″N	2,179–2,202 m	38–42%	Calcareous soil	0.71	Herbaceous plants: mainly Trifolium Linn, Juncus effuses and Artemisia carvifolia.
Vegetation restoration (VR)	104°13′53.40″–104°13′7.0″E 26°51′56.86″–26°52′0.81″N	2,179–2,202 m	55–60%	Calcareous soil	0.68	Arbor community: mainly planted *Populus yunnanensis* Dode; shrub community: *Zanthoxylum piperitum*; herbaceous plants: Coreopsis drummondii and Artemisia carvifolia, *etc*.

From top to bottom, five sampling points mountaintop (MT), hillside (MO), foothills (MF), shore (SH) and wetland (WL) in each sample belt. A total of 180 samples were collected from 13 sample points through “five-point method”, and the depth of the sampling profile was 0–50 cm with a 10 cm depth interval (*i.e*., 0, 10, 20, 30, 40 and 50 cm). Subsequently, soil samples collected in the sample site were fully mixed after removing impurities, and the final soil samples were obtained. The soil samples were divided into two parts and sealed in self-sealed bags and marked and stored at low temperature. One soil sample is used for the determination of heavy metals in soil, and the other is used to determine carbon, nitrogen, phosphorus, pH, moisture content, bulk density, electrical conductivity and other indexes.

### Pretreatment and determination of samples

Soil organic carbon (SOC), total nitrogen (TN), and total phosphorus (TP) were measured as described previously (Walkley & Black, 1934). Nitrate nitrogen (NO_3_^−^-N), ammonium nitrogen (NH_4_^+^-N), soil organic carbon (SOC), readily oxidizable carbon (ROC), dissolved organic carbon (DOC), pH, soil bulk density (BD), electrical conductivity (EC) and soil moisture (SWC) were measured as described previously (Wu et al., 2022a), Chemicals, including sulfuric acid, hydrochloric acid, sodium hydroxide, were purchased from Sinopharm Chemical Reagent Co., Ltd with reagent grade or higher purity.

Concentrations of Cr, Ni, As, Zn, Pb, iron (Fe), manganese (Mn) and calcium (Ca) were determined by DELTA Professional-Handheld XRF analyzers (Olympus Corporation, Shinjuku City, Tokyo, Japan). Soil aggregates were determined by wet sieve method, and the classified through the National Forestry and Grassland Administration of China (1987). The physical and chemical properties of the soil are listed in Table 2.

**Table 2:** Physical and chemical properties of surface soil from the different transect.

Transect	Non-karst					Karst					Vegetation restoration		
	MT	MO	MF	SH	WL	MT	MO	MF	SH	WL	MT	MO	MF
BD	1.41 ± 0.11a	1.39 ± 0.02a	1.36 ± 0.14a	1.51 ± 0.02a	1.32 ± 0.02a	1.37 ± 0.06a	1.30 ± 0.09a	1.26 ± 0.09a	1.49 ± 0.18a	1.48 ± 0.17a	1.59 ± 0.19a	1.22 ± 0.06a	1.41 ± 0.16a
SWC/%	25.15 ± 6.57b	27.56 ± 3.65b	30.87 ± 4.27ab	26.62 ± 1.57b	36.95 ± 0.93a	22.02 ± 0.19a	29.06 ± 0.06a	25.42 ± 0.16a	30.67 ± 0.18a	29.28 ± 0.17a	31.66 ± 0.06a	37.61 ± 0.09a	38.67 ± 0.09a
pH	4.19 ± 0.11b	4.41 ± 0.29b	4.44 ± 0.23b	7.40 ± 0.30a	7.16 ± 0.43a	8.26 ± 0.10a	8.22 ± 0.09a	7.77 ± 0.08a	7.81 ± 0.13a	7.82 ± 0.13a	7.99 ± 0.10a	6.47 ± 0.33a	7.38 ± 0.61a
EC/μs·cm^−3^	42.77 ± 12.23c	38.87 ± 15.42c	36.03 ± 11.44c	75.90 ± 15.40b	125.63 ± 17.25a	137.37 ± 13.69b	81.10 ± 24.47cd	59.70 ± 18.93d	116.67 ± 8.61bc	214.03 ± 34.70a	99.20 ± 32.72a	36.63 ± 11.87a	89.83 ± 47.55a
SOC/g·kg^−1^	16.59 ± 2.17ab	18.07 ± 3.35a	11.16 ± 4.32b	18.57 ± 1.49a	19.47 ± 1.26a	8.77 ± 1.76c	5.84 ± 1.29cd	11.50 ± 2.29d	19.81 ± 0.63b	26.52 ± 3.57a	12.36 ± 1.43c	9.37 ± 1.51cd	3.03 ± 0.91d
ROC/mg·kg^−1^	22.42 ± 3.11a	22.38 ± 1.47a	23.91 ± 5.92a	23.48 ± 3.12a	21.61 ± 1.32a	15.85 ± 4.94ab	8.54 ± 1.94ab	12.30 ± 4.98c	23.78 ± 7.02a	14.58 ± 1.01ab	16.48 ± 4.02ab	17.00 ± 4.82ab	11.89 ± 3.90b
DOC/mg·kg^−1^	2.47 ± 0.71a	2.14 ± 0.28a	2.26 ± 0.22a	2.03 ± 0.34a	2.64 ± 0.10a	1.58 ± 0.11ab	1.74 ± 0.11ab	1.26 ± 0.07b	1.33 ± 0.17b	1.96 ± 0.47a	1.99 ± 0.47a	1.80 ± 0.46a	1.35 ± 0.24a
TN/g·kg^−1^	1.81 ± 0.46a	1.55 ± 0.36a	1.57 ± 0.21a	2.00 ± 0.16a	2.28 ± 0.7a	0.99 ± 0.23b	0.92 ± 0.08b	0.91 ± 0.11b	1.93 ± 0.46a	1.67 ± 0.41a	1.44 ± 0.04ab	1.54 ± 0.41a	1.03 ± 0.08b
NH_4_^+^-N/mg·kg^−1^	14.81 ± 1.04a	3.19 ± 0.82a	4.73 ± 1.14a	7.51 ± 3.17a	7.43 ± 1.82a	2.81 ± 0.61b	3.19 ± 0.82b	4.73 ± 1.14ab	7.51 ± 3.17a	7.43 ± 1.82a	3.44 ± 0.76a	5.21 ± 0.13a	3.98 ± 1.13a
NO_3_^−^-N/mg·kg^−1^	4.24 ± 0.33a	6.29 ± 2.33a	6.49 ± 1.35a	5.30 ± 1.14a	3.59 ± 0.28a	1.08 ± 0.15c	1.34 ± 0.89c	1.44 ± 0.35c	7.12 ± 1.17a	4.41 ± 1.52b	2.61 ± 0.07a	3.17 ± 1.36a	2.70 ± 0.73a
TP/mg·kg^−1^	2.81 ± 0.65c	3.11 ± 2.26b	2.61 ± 1.05ab	5.94 ± 1.24c	5.85 ± 1.51c	5.98 ± 0.73ab	5.56 ± 1.90bc	4.22 ± 1.24bc	8.13 ± 0.88a	3.61 ± 1.24c	1.91 ± 1.45b	6.51 ± 0.45a	6.12 ± 0.07a
AP/mg·kg^−1^	4.14 ± 1.26ab	5.60 ± 0.65a	6.13 ± 1.24a	4.12 ± 1.90ab	1.77 ± 0.07b	1.93 ± 1.51a	1.40 ± 1.05a	2.58 ± 2.26a	5.62 ± 0.80b	5.62 ± 0.88b	2.38 ± 0.73a	6.65 ± 1.45a	6.11 ± 0.45ab
Fe/g·kg^−1^	0.016 ± 0.003d	0.018 ± 0.003cd	0.024 ± 0.001bc	0.026 ± 0.005b	0.036 ± 0.001a	0.033 ± 0.000ab	0.037 ± 0.004a	0.036 ± 0.008a	0.034 ± 0.003a	0.022 ± 0.005b	0.031 ± 0.006a	0.034 ± 0.003a	0.032 ± 0.002a
Mn/mg·kg^−1^	112.59 ± 34.88b	275.79 ± 146.17ab	295.33 ± 77.24ab	372.33 ± 101.66a	414.56 ± 29.68a	336.78 ± 26.31ab	422.22 ± 63.26a	324.89 ± 40.14ab	484.33 ± 76.38a	231.78 ± 32.42b	294.56 ± 89.65b	603.44 ± 64.19a	508.56 ± 47.91a

**Note:**

Mean ± SD, same below; MT, mountaintop; MO, hillside; MF, foothills; SH, shoreside; WL, wetland; BD, soil bulk density; SMC, soil moisture content; pH, pH value; EC, electrical conductivity; SOC, soil organic carbon; ROC, readily oxidizable carbon; DOC, dissolved organic carbon; TN, total nitrogen; NH_4_^+^-N, ammonium nitrogen; NO_3_^−^-N, Nitrate nitrogen; TP, total phosphorus; AP, available phosphorus; Fe, iron; Mn, manganese; lowercase letters represent significant differences in soil physical and chemical properties with the same point (*p* < 0.05).

### Data analysis and statistics

The data were analyzed by Excel 2010 and SPSS 26. Redundancy analysis (RDA) of the heavy metals content and physicochemical properties of the soil was performed by Canoco software (version 5.0; http://www.canoco5.com/). All graphics were plotted in Origin 9.1 (https://www.originlab.com/index.aspx?go=Support&PID=2131).

## Results

### Distribution characteristics of soil aggregates under different landforms

**Figure 2:**
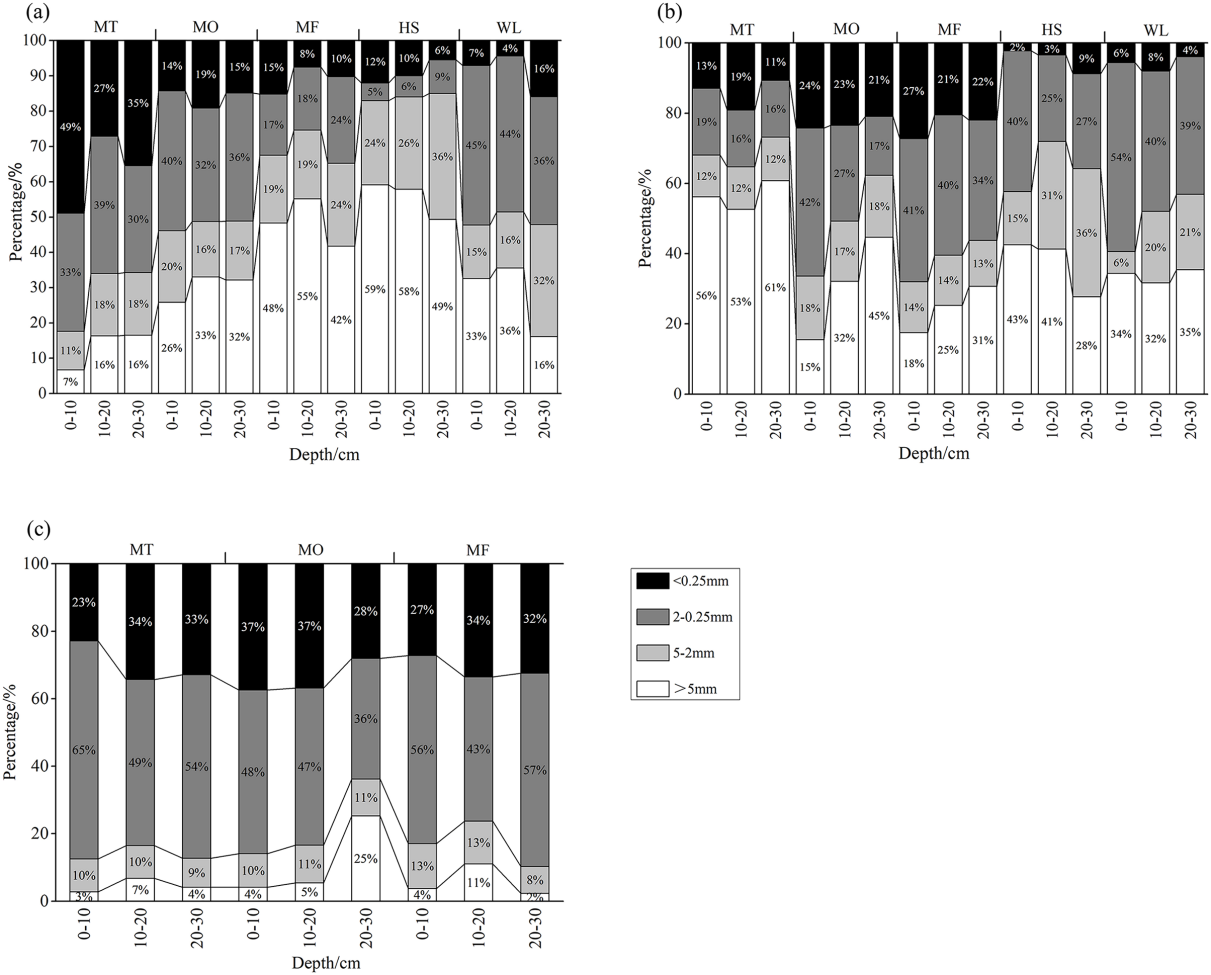
(A–C) Distribution of soil aggregates in vegetative restoration. MT, mountaintop; MO, hillside; MF, foothills; SH, shoreside; WL, wetland.

As shown in Fig. 2, soil aggregates shows different distribution characteristics in three landforms. In non-karst geomorphology, the proportion of soil aggregates larger than 2 mm increased gradually from MT to MF and gradually occupied the dominant position, while the proportion of 0.25–2 mm soil aggregates decreased slowly. In karst landform without vegetation restoration, although the proportion of soil aggregates larger than 2 mm decreases from MT to MF, and the proportion of 0.25–2 mm soil aggregates shows the opposite trend with non-karst geomorphology, indicating that soil aggregates and microaggregates are easy to be lost under karst landforms. In addition, more than 50% of soil aggregates in SH and WL sample sites are mainly larger than 2 mm soil aggregates, accounting for more than 50%, and the proportion of soil aggregates of each particle size changes little with the increase of depth.

However, the distribution of soil aggregates after vegetation restoration is different from non-karst geomorphology and karst landform without vegetation restoration. The proportion of surface soil 0.25–2 mm aggregates in vegetation restoration belt increased to more than 40% especially larger than 2 mm (13–17%). The proportion of surface soil ≤2 mm aggregates was also increased to 83% and 88%, respectively. The distribution proportion of soil aggregates in each sample site of vegetation restoration was similar and the change of depth is not obvious. The stable proportion of surface soil 0.25–5 mm aggregates accounted for more than 90%. This result indicated that vegetation restoration significantly improved the soil structure. Previous studies has shown the similar phenomenon, that is, the content of organic carbon in soil aggregates is higher in the range of 5–0.25 mm particle size, and the increase of organic carbon content is helpful to the formation of soil water-stable aggregates as well as change the soil structure and properties (Xu et al., 2017; Cheng et al., 2013). In addition, the changed soil structure could also attributed to the increased vegetation coverage, which increased plant roots and further improved the ability to fix soil.

Furthermore, this study showed that the content of soil organic carbon is not always in a steady upward trend in the early stage of vegetation restoration, and the frequent export and input of soil organic carbon leads to great fluctuation (Table 2), which is consistent with the results of previous studies (Xie et al., 2015; Zhang et al., 2020b). The increase of soil organic matter content is beneficial to the oxidizable transformation and enrichment of heavy metals (Zhang & Han, 2022).

### Distribution characteristics of soil heavy metal contents in non-karst and karst landforms

**Figure 3:**
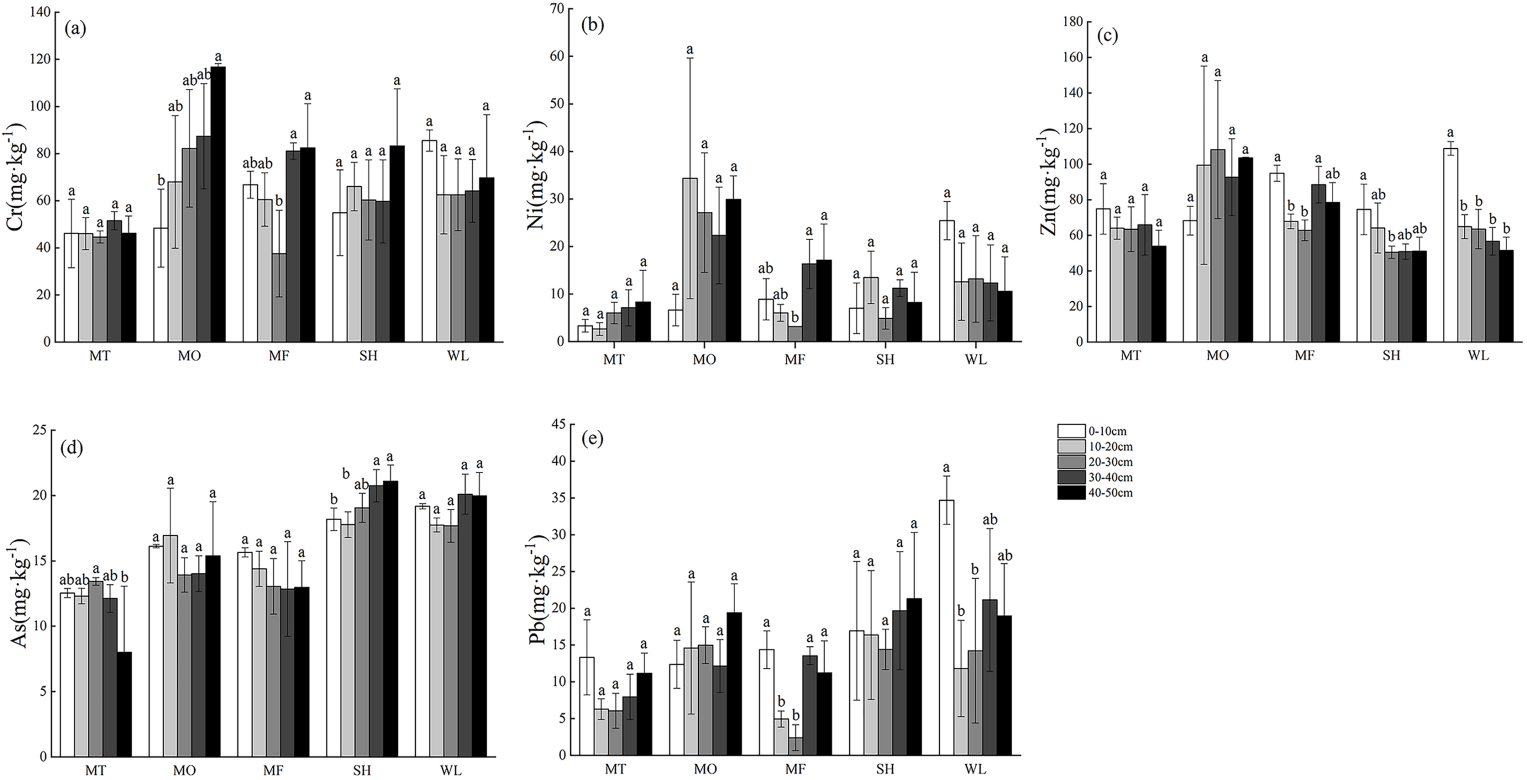
(A–E) Profile distribution of heavy metals in soils in non-karst landforms. MT, mountaintop; MO, hillside; MF, foothills; SH, shoreside; WL, wetland. Lowercase letters represent significant differences in soil heavy metal contents at different depths at the same point (*p* < 0.05).

The distribution of heavy metals (Cr, Ni, Zn, As and Pb) in non-karst soil profile (0–50 cm) is shown in Fig. 3. The contents of Cr, As and Pb in the soil of SH and WL increased with the increase of soil depth, while the contents of Ni and Zn in the surface and bottom layer were higher than those in the middle layer, showing a “V” type. Except for As, other heavy metals in WL were enriched in the surface layer, and the contents of Zn and Pb were significantly different from those in other soil profiles (*p* < 0.05). As a whole, the content of heavy metals in soil of MO was higher than that of MT and MF as a whole, and the content of heavy metals increased gradually along the soil profile, in which Cr showed significant difference in profile (*p* < 0.05). The content of heavy metals in MF soil showed a tipping point in 20–30 cm profile in MF was the lowest, and it decreased in 0–20 cm while increased in 30–50 cm profile. Each profile showed significant difference (*p* < 0.05). There is no clear trend for the content of heavy metals in each sample site and profile did not follow the obvious rule.

**Figure 4:**
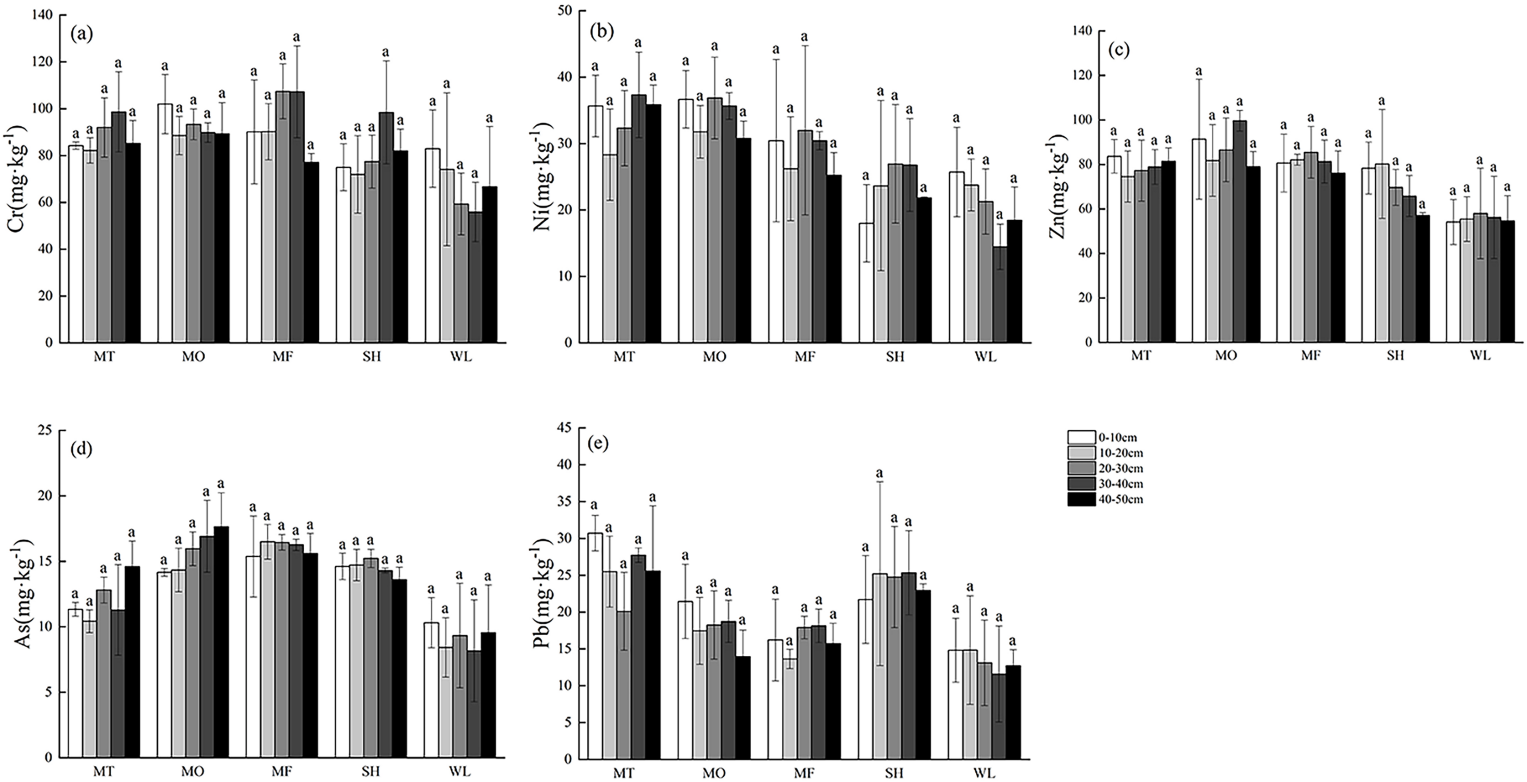
(A–E) Profile distribution of heavy metals in soils in karst landforms. MT, mountaintop; MO, hillside; MF, foothills; SH, shoreside; WL, wetland. Lowercase letters represent significant differences in soil heavy metal contents at different depths at the same point (*p* < 0.05).

The profile distribution of heavy metals in karst soil without vegetation restoration is shown in Fig. 4. Four kinds of heavy metals (except As) in the sample belt show a decreasing trend from MT to WL. MT, MO and MF have different degrees of enrichment of heavy metals in the surface layer of soil, and the content of heavy metals reaches the lowest in 10–20 or 20–30 cm layer, and then increases, showing different degrees of “V” type. The content of heavy metal as increased with the increase of soil depth at MT and MO, but there was no significant difference. The content of five heavy metals at SH was higher than that at WL. There was no significant difference in the content of heavy metals in soil profiles among different sample sites (*p* > 0.05), and there was also no significant difference in numerical range.

### Distribution characteristics of soil heavy metal content in vegetation restoration sample zone

**Figure 5:**
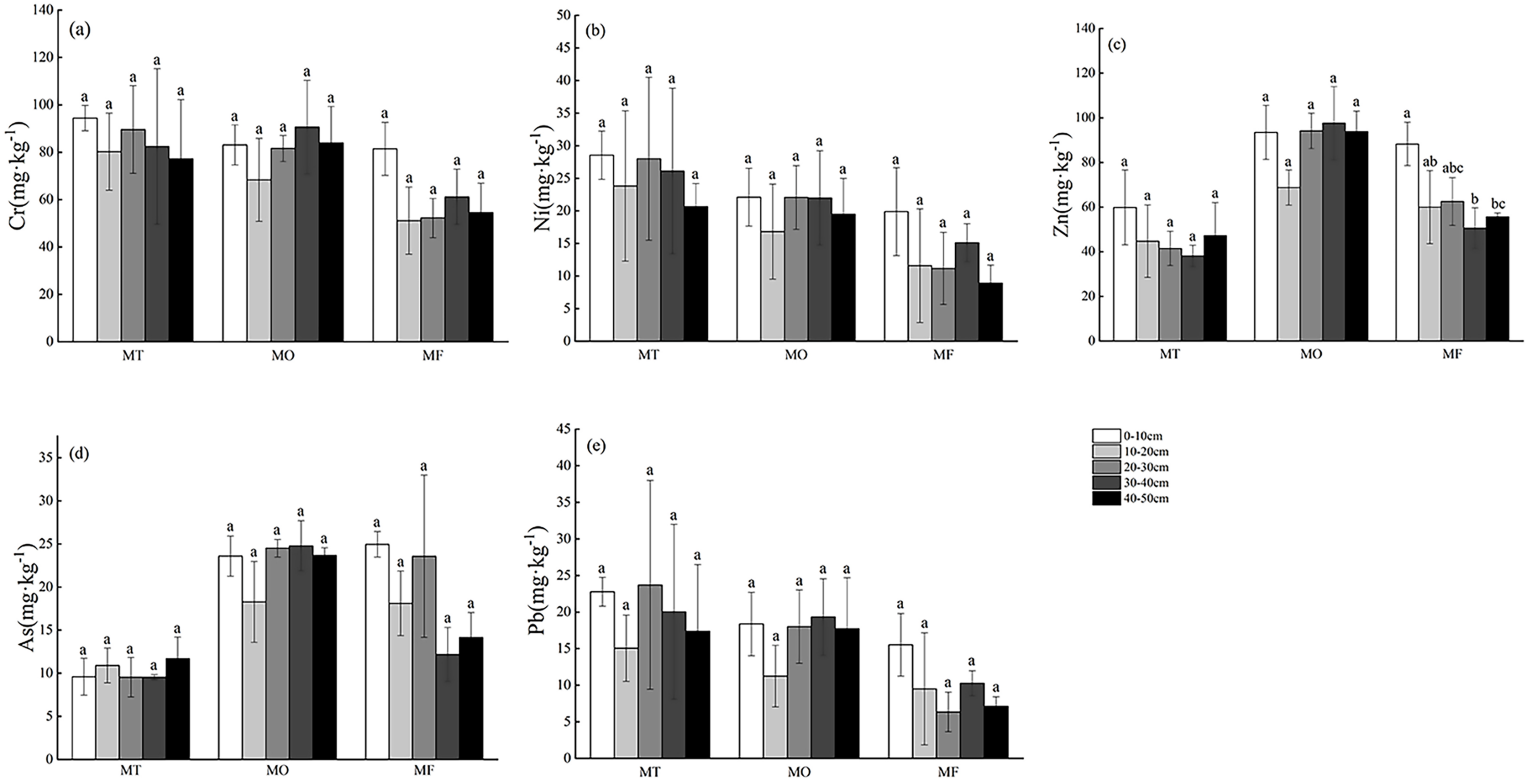
(A–E) Profile distribution of heavy metals in soil of vegetation restoration landforms. MT, mountaintop; MO, hillside; MF, foothills; SH, shoreside; WL, wetland. Lowercase letters represent significant differences in soil heavy metal contents at different depths at the same point (*p* < 0.05).

The distribution of heavy metals in soil profile of vegetation restoration was shown in Fig. 5. Heavy metals Cr, Ni and Pb gradually decreased gradually from MT to MF. The lowest value reached at 10–20 or 20–30 cm in soil profile, and accumulated in surface and deep soil (20–50 cm). In sample sites MT, MO and MF, the distribution trend of heavy metals in soil profile was basically the same, and showed different degrees of surface enrichment. The soil heavy metals in each profile were the same as those in the karst zone without vegetation restoration, and there was no significant difference (*p* > 0.05).

### Correlation analysis between heavy metal content and physicochemical factors in topsoil

#### Principal coordinate analysis (PCA) of soil physical and chemical properties in different geomorphological landforms

**Figure 6:**
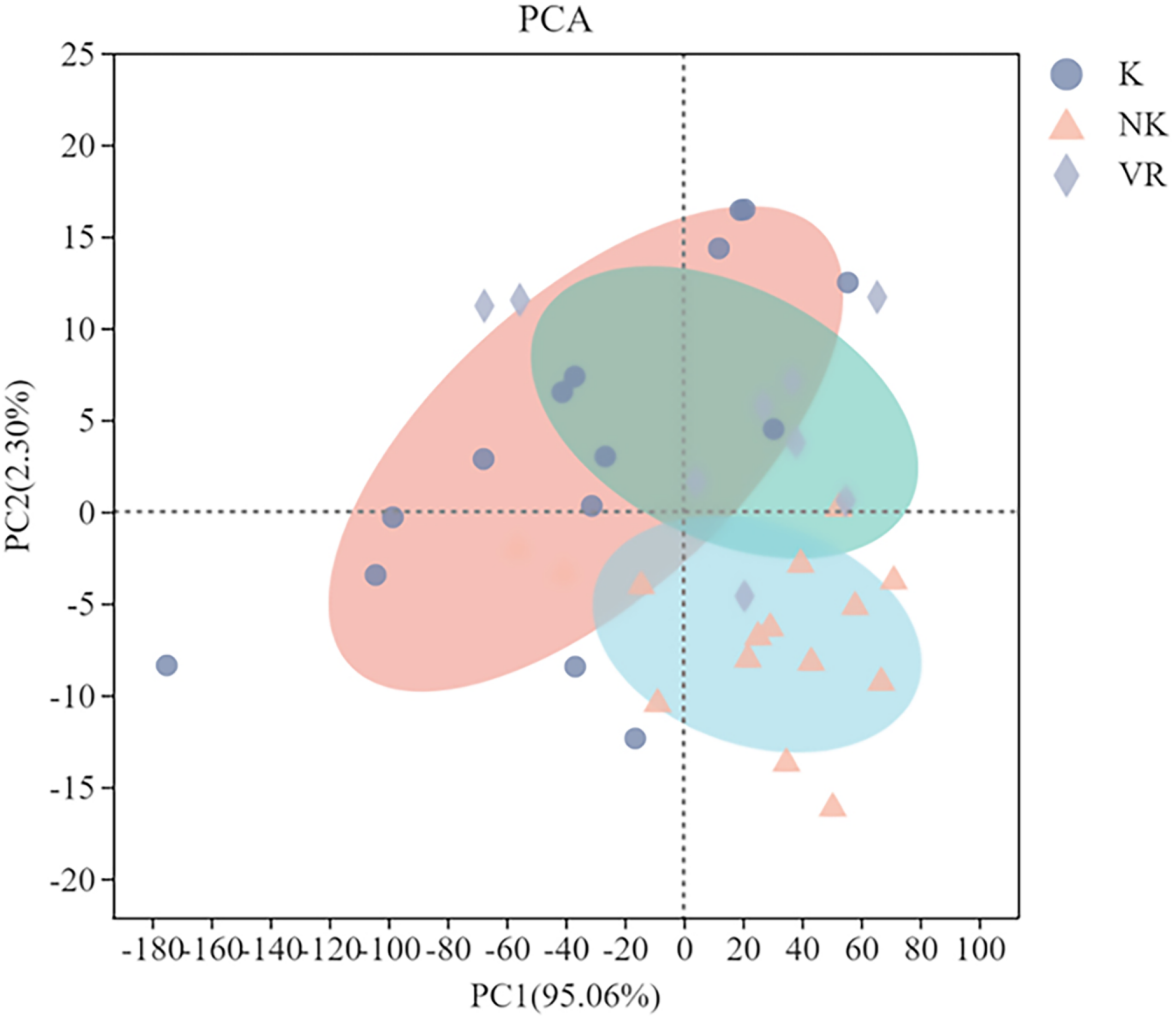
Principal coordinate analysis of soil physical and chemical properties chemical properties. PCA, The principal component analysis; K, Karst; NK, Non-karst; VR, vegetation restoration.

The principal component analysis (PCA) of the soil physical and chemical properties under three sample bands is shown in Fig. 6. In every zone, soil properties were mainly controlled by two principal coordinate (PC) components. There was a cumulative variance of 97.36% among them with PC1 explaining 95.06% and PC2 explaining 2.30%. The overlap area of vegetation restoration landform and karst landform was large, which showed that the soil physical and chemical properties of the two were similar, but the overlap area of non-karst landform was smaller, indicating that there was a great difference between non-karst landform soil physical and chemical properties and the two.

#### linear stepwise regression analysis

According to previous study (Golhar, Choudhari & Patil, 2021; Wu et al., 2022b), soil factors and path analysis have a certain correlation. Hence, linear stepwise regression analysis is selected to reflect the effect of environmental factors on soil heavy metals. Through screening and analysis, the statistical regression models of heavy metals and other soil physical and chemical factors in non-karst, karst and vegetation remediation soils are as follows:

Non-karst:

Cr = 46.344 + 0.004 Fe − 6.348 pH − 2.381 As

Ni = −7.829 + 0.681 Pb + 0.018 Mn

Zn = 16.597 + 0.004 Fe −8.609 pH + 0.536 NH_3_^+^-N

As = 12.447 + 1.459 TP + 0.086 Ni − 1.828 TN

Pb = 6.531 + 0.749 Ni + 0.003 Ca

Karst:

Cr = 56.791 + 0.992 Ni

Ni = 16.454 − 2.273 NH_3_^+^-N + 0.283 Cr

Zn = 9.619 + 0.002 Fe

As = 3.554 + 0.001 Fe − 0.185 Pb + 0.007 Mn

Pb = −22.883 + 0.119 Zn + 25.194 BD − 0.183 NH_3_^+^-N + 5.102 TN

Vegetation restoration:

Cr = 121.420 − 6.638 AP

Ni = −3.608 + 1.324 Pb − 13.111 TN + 1.948 pH

Zn = 24.486 + 0.120 Mn

As = 8.918 + 2.840 TP − 0.468 SOC

Pb = −29.383 + 0.279 Ni + 30.459 BD + 0.182 ROC − 0.046 As

It can be seen from Table 3 that the contents of five kinds of heavy metals in non-karst soils are affected by soil physical and chemical factors and other metals. The direct path coefficient of Fe element content in soil to Cr element content is greater than its indirect path coefficient, indicating that the main contribution to Cr content is direct and positive effect. Among all soil factors, Fe element content in soil has the greatest indirect effect on Cr content. It has an indirect positive effect on Cr mainly through pH and the contents of As in soil. The direct path coefficient of Pb to Ni content is larger than its indirect path coefficient, suggesting the main contribution is direct and positive effect, while the contribution of Mn element content in soil to Ni element content is indirect. However, the correlation coefficient between Mn and Ni content is larger suggesting Mn is also a main factors. Similarly, the direct path coefficient of Fe element content in soil on Zn element content is larger than its indirect path coefficient, and its main contribution was direct effect, both showed positive effect, while pH and NH_4_^+^-N have the greatest indirect effect on Zn content, mainly through the indirect effect on Fe element. The direct effect of soil TP on heavy metal As content is larger than indirect effect, and shows positive effect, while TN and Ni element show opposite effect, their indirect path coefficient is larger, mainly show indirect effect; the direct path coefficient of Ni element content in soil on heavy metal Pb element content is larger than its indirect path coefficient, while soil Ca content shows indirect effect, and the correlation coefficients between them and Pb are larger.

**Table 3:** Simple correlation coefficient decomposition of soil heavy metals and soil factors in non-karst landforms.

Heavy metal	Target	Coefficient of association	Direct bore coefficient	Indirect diameter coefficient										Decision coefficient R^2^
				pH	TN	NH_4_^+^-N	Ni	As	Pb	Fe	Mn	Ca	TP	
Cr	Fe	0.908	1.440	−0.303				−0.229						0.973
	pH	0.436	−0.408					−0.225		1.068				
	As	0.550	−0.285	−0.322						1.158				
Ni	Pb	0.917	0.754								0.163			0.893
	Mn	0.718	0.281						0.437					
Zn	Fe	0.803	1.517	−0.441		−0.272								0.911
	pH	0.337	−0.595			−0.194				1.126				
	NH_4_^+^-N	−0.241	0.438	0.263						−0.942				
As	TP	0.904	0.859		−0.121		0.167							0.941
	Ni	0.689	0.329		−0.075								0.436	
	TN	0.236	−0.260				0.095						0.401	
Pb	Ni	0.917	0.677									0.240		0.897
	Ca	0.819	0.338				0.481							

**Note:**

Cr, chromium; Ni, nickel; As, arsenic; Zn, zinc; Pb, lead; pH, pH value; TN, total nitrogen; NH_4_^+^-N, ammonium nitrogen; TP, total phosphorus; Fe, iron; Mn, manganese; Ca, calcium.

The contents of five kinds of heavy metals in karst soil are listed in Table 4. It can be seen that, the contents are affected by soil physical and chemical properties and other metals. The main contribution of Ni element content in soil to Cr element content and Fe element content in soil to Zn element content is direct effect, only one factor has significant effect, and all of them show positive effect. The indirect path coefficient of soil NH_4_^+^-N to Ni element content is larger than its direct path coefficient, indicating that its main contribution to Ni content is the indirect effect by affecting other factors, while the direct path coefficient of Cr element content in soil to heavy metal Ni is larger than its indirect path coefficient, indicating that its main contribution is direct effect.

**Table 4:** Simple correlation coefficient decomposition of soil heavy metals and soil factors in karst landforms.

Heavy metal	Target	Coefficient of association	Direct bore coefficient	Indirect diameter coefficient								Decision coefficient R^2^
				BD	TN	NH_4_^+^-N	Cr	Zn	Pb	Fe	Mn	
Cr	Ni	0.605	0.605									0.366
Ni	NH_4_^+^-N	−0.688	−0.573				−0.115					0.675
	Cr	0.605	0.464			0.142						
Zn	Fe	0.843	0.843									0.711
As	Fe	0.858	0.969						−0.295		0.184	0.928
	Pb	0.218	−0.492							0.581	0.128	
	Mn	0.685	0.298						−0.212	0.600		
Pb	BD	0.512	0.697		−0.021	−0.173		0.009				0.898
	NH_4_^+^-N	−0.512	−0.621	0.194	0.094			−0.180				
	TN	0.019	0.281	−0.052		−0.207		−0.002				
	Zn	0.689	0.315	0.021	−0.002	0.354						

**Note:**

Cr, chromium; Ni, nickel; As, arsenic; Zn, zinc; Pb, lead; BD, Bulk density; TN, total nitrogen; NH_4_^+^-N, ammonium nitrogen; Fe, iron; Mn, manganese.

The direct path coefficient of soil Fe element content in soil to heavy metal As element content is larger than its indirect path coefficient, indicating that the main contribution to As element content is direct effect and positive effect, while the indirect path coefficient of Pb element and Mn element is larger than their direct path coefficient, indicating that their main contribution to As element content is the indirect effect by affecting other factors. The correlation between soil factors and As content is Fe > Mn > Pb. The direct path coefficients of soil BD, NH_4_^+^-N and TN to heavy metal Pb content are larger than their indirect path coefficients, indicating that the main contribution to Pb content is direct effect, in which BD shows the largest positive effect, followed by TN, while NH_4_^+^-N show negative effect, and the main contribution of Zn element content in soil to As element content is indirect effect by affecting other factors. The correlation between soil factors and Pb element content is Zn > BD = NH_4_^+^-N > TN.

From Table 5, it can be seen that the main contribution of AP to Cr element content and Mn element content in soil to Zn element content in vegetation restoration soil is direct effect, in which AP has negative effect on heavy metal Cr element content, while Mn element content in soil has positive effect on heavy metal Zn. The direct effect of soil Pb and TN on the content of heavy metal Ni is greater than the indirect effect, and Pb element content has a positive effect on Ni element content, and vice versa, while the main contribution of pH to Ni content is the indirect effect by affecting other factors, and the correlation between soil factors and Ni content show that Pb is the largest and pH is the smallest. The direct path coefficient of soil TP and SOC content to heavy metal As is larger than its indirect path coefficient, indicating that the main contribution to As content is direct effect, and TP shows positive effect, SOC vice versa. Soil BD had a direct effect on heavy metal Pb content, and the indirect path coefficient of Ni element content in soil, ROC and As element content in soil to heavy metal Pb content is greater than their direct path coefficient, indicating that the main contribution of soil factors to Pb content is indirect effect by affecting other factors, and the correlation between soil factors and Pb content is Ni > BD > As > ROC.

**Table 5:** Decomposition of simple correlation coefficients between heavy metals and soil factors in vegetative remediation soils.

Heavy metal	Target	Coefficient of association	Direct bore coefficient	Indirect diameter coefficient									Decision coefficient R^2^
				BD	pH	SOC	ROC	TN	Ni	As	Pb	TP	
Cr	AP	−0.878	−0.878										0.772
Ni	Pb	0.950	0.998		−0.033			−0.015					0.988
	TN	−0.183	−0.269		0.033						−0.054		
	pH	−0.055	0.180					−0.049			−0.185		
Zn	Mn	0.912	0.912										0.832
As	TP	0.981	0.838			0.096							0.967
	SOC	−0.776	−0.322									−0.250	
Pb	Ni	0.950	0.370	0.467			0.084			0.029			0.999
	BD	0.920	0.588				0.014		0.294	0.024			
	ROC	0.404	0.186	0.044					0.168	0.006			
	As	−0.444	−0.070	−0.203			−0.017		−0.153				

**Note:**

Cr, chromium; Ni, nickel; As, arsenic; Zn, zinc; Pb, lead; BD, Bulk density; pH, pH value; SOC, Organic carbon; ROC, Readily oxidized organic carbon; TN, Total nitrogen; TP, total phosphorus; AP, available phosphorus.

Generally speaking, non-metallic soil factors such as BD, TN, NH_4_^+^-N, TP and AP play a direct role in the three transects, while pH plays a role by affecting other factors in non-karst soils. The determination coefficient (R^2^) of the linear stepwise regression analysis equation for Pb content in vegetation restoration belt is 0.999, and the path coefficient of residual factors is 0.045, which indicates that other factors have little influence on Pb content. But the path coefficient e values of other soil heavy metal contents are relatively large (e_NK-Cr_ = 0.231; e_NK-Ni_ = 0.450; e_NK-Zn_ = 0.412; e_NK-As_ = 0.338; e_NK-Pb_ = 0.442; e_K-Cr_ = 0.931; e_K-Ni_ = 0.738; e_K-Zn_ = 0.703; e_K-As_ = 0.373; e_K-Pb_ = 0.440; e_VR-Cr_ = 0.774; e_VR-Ni_ = 0.154; e_VR-Zn_ = 0.555; e_VR-As_ = 0.412) indicates that there are other factors that have great influence on the content of heavy metals in soil in different zones, which need to be further studied.

## Discussion

### Influence of distribution characteristics of heavy metals in karst geomorphology soil

The content of heavy metals in soil is closely related to soil parent material, soil physical and chemical properties and microorganisms, and is also affected by human activities (Li et al., 2022). Based on the results obtained in Figs. 3 and 4, it was appeared that the content and distribution of heavy metals in the soil profile between the non-karst zone and the karst zone are markedly different. In non-karst zone, heavy metals enriched in the surface soil and then decreased in 0–10 cm, while increased in the range of 10–50 cm. In contrast, only Ni and Pb showed this tendency in the karst zone. These phenomena can be attributed to the following reasons. On the other hand, the rich organic complexes in Arbor forest soil have strong adsorption and chelation, thus inhibiting the migration of heavy metals (Zhang et al., 2019). Moreover, the forms of heavy metals are mainly composed of stable residues, which can be stable in sediments for a long time. Therefore, the heavy metals remain in the middle and upper layers of the soil, and finally enriched in the surface layer (Jitendra, Rachna & Naveen, 2017). On the other hand, a large number of microorganisms could grow in the forest ecosystem with the developed roots and abundant litter, and heavy metals may be adsorbed on extracellular polymers or cell wall components produced by microorganisms, which can regulate the concentration of heavy metals in the soil (Ma et al., 2014; Zhao et al., 2015).

In addition, the contents of Cr, Ni, Zn, As and Pb in Caohai area detected in present study were lower than or close to the background contents of soil heavy metals in Guizhou Province (Wang et al., 2021). Some studies have reported that when the lower levels of heavy metals in soil had no or little effect on the normal metabolic activities of plants, plants could increase the number of rhizosphere soil microorganisms and enzyme activities, secrete H^+^ to reduce rhizosphere soil pH value (Zeng et al., 2008), increase the bioavailability of mineral nutrients and heavy metals in the root zone by regulated root secretions, and thus promote the absorption and utilization of heavy metals by plants (Gai et al., 2020). At the same time, the migration ability of heavy metals in the acidic non-karst soil environment (pH < 5) will also enhance under the action of leaching, resulting in heavy metals were precipitated in bottom and enrichment (Tang et al., 2021).

In summary, the common distribution characteristic of heavy metals in non-karst soils is surface and bottom precipitation enrichment, while that of karst soils are affected by surface vegetation, root exudates, microorganisms and leaching, and leading to the different distribution characteristic.

### Effect of soil heavy metal profile distribution characteristics in vegetation restoration sample zone

Compared with karst soil, the contents of heavy metals Cr, Ni and Pb in vegetation restoration sample zone were decreased in a lesser extent. This may be ascribed to heavy metals in the soil were absorbed by plants. In previous study (Qi et al., 2022), it was found that the dominant herbaceous plants in native *Compositae* and *Gramineae* had strong enrichment and transport ability to heavy metals such as Cd, Cr and Ni. Moreover, the contents of heavy metals in the surface layer of soil were higher than those in 10–20 cm layer. The vegetation in the vegetation restoration sample zone is mainly covered by dwarf trees, shrubs and herbaceous vegetation communities, and the herbaceous communities take *Chrysanthemum morifolium* and *Artemisia annua* as the dominant species in the vegetation restoration area, and plants absorb heavy metals in the soil. Reduce the mass fraction of heavy metals (Li et al., 2005), which is also an important factor that the heavy metals in the surface soil are higher than those in the middle layer.

In this study, it was found that ROC, DOC and inorganic nitrogen in vegetation remediation soil were significantly higher than those in karst soil. The research of Xie et al. have clarified the vegetation restoration could not only strengthen the water retention ability of soil, but also change the dynamic balance of various elements, which reducing soil organic carbon mineralization and soil erosion to a certain extent. Therefore, soil carbon sequestration capacity gradually enhanced, and then the carbon content in vegetation remediation soil were significantly increased. Except the change of carbon content in soil, pH was also decreased (0.2–0.4). This phenomenon maybe caused by the H^+^ secreted from plants, and the root exudates produced by plant further promoting the growth of soil microorganisms. The reduced pH demonstrated that vegetation restoration can enhance soil microbial activity significantly.

In addition, heavy metal content is enriched on the soil surface and deep layers, which will be attributed to the following points. Firstly, there is competition between plant adsorption and microbial biosorption for heavy metals (Ma et al., 2014; Zhao et al., 2015; Ma et al., 2010). Some research have proved that soil pH and DOC are important factors affecting the bioavailability of heavy metals (Sizmur & Hodson, 2009), which can transform residual heavy metals into highly mobile forms in soil, and further increase bioavailability (Zhang et al., 2019; Xie, Ren & Li, 2007, Zhang et al., 2020a). The absorption process of heavy metals by plants would be affected by root exudates, which have the effect on the pH value, occurrence and solubility of heavy metals in rhizosphere soil (Wang et al., 2022a). For example, organic acids and metal complexes play an important role in the mechanism of heavy metal absorption in roots by increasing the concentration of free metal ions (Lin et al., 2022). Secondly, although vegetation restoration increases vegetation coverage and soil water retention capacity, it is still in the early stage of vegetation restoration. Study has reported that in many kinds of heavy metals are still in residual form different degrees of rocky desertification soil, (Xie, Ren & Li, 2007), so the content of heavy metals in soil is further leached by surface runoff. Therefore, the content of heavy metals shows a deep aggregation (soil depth ≥ 30 cm).

## Conclusion

1) The linear stepwise regression analysis showed that non-metallic soil factors containing BD, TN, NH4^+^-N, TP, and AP mostly directly affect the heavy metals content in the soil of the three transects. The contents of As and Pb in the vegetation remediation transect were also affected by SOC and ROC, and the above all factors profoundly affect the path of plant absorption of heavy metals. In addition, the acidic condition (pH < 5) is benefit for enhancing the bioavailability and migration capacity of heavy metals, and more heavy metals were absorbed and utilized by plants. The distribution characteristics of heavy metals appeared in non-karst soils are surface enrichment and bottom sedimentation enrichment.
2) Compared with karst landforms, vegetation restoration increased the proportion of surface soil ≤2 mm aggregates. The proportion of topsoil 0.25–5 mm aggregates was more than 90%. The ability of roots to fix soil is further reinforced. The increased soil organic carbon content of 0.25–5 mm aggregates was benefit for the formation of water-stable aggregates, and then changed the soil structure and properties.
3) Through vegetation remediation, the material cycle in topsoil is enhanced. The uptake efficiency of plants, the bioadsorption of microorganisms, and the coupling effect of root exudates were further strengthened. These variation in vegetation remediation soil could lead to the slightly lower contents of Cr, Ni and Pb, the increase of soil organic matter content (especially dissolved organic carbon) and the decrease of soil pH value. Besides, a part of heavy metals in soil were transformed into the forms with highly mobile, while residual heavy metals were further leached by surface runoff.

Briefly, in the present work, the distribution characteristics of non-karst soil, karst soil and karst soil with vegetation restoration were analyzed. Some factors that affecting the distribution characteristics, migration and transformation of heavy metals in karst soil under vegetation restoration were explored, and the main methods to reduce the content of heavy metals in karst soil were investigated. Phytoremediation can improve the content of organic matter in heavy metal contaminated soil and the stability of soil aggregates, improve soil structure and enrich heavy metals to the surface layer. It was concluded that the method of plant activation and enrichment extraction can be widely used in the remediation of heavy metal contaminated soil in Caohai area of Guizhou and other karst area.

## Supplemental Information

10.7717/peerj.15044/supp-1 Supplemental Information 1 Soil physicochemical properties.

10.7717/peerj.15044/supp-2 Supplemental Information 2 Soil heavy metal data.

## Additional Information and Declarations

### Data Availability

The following information was supplied regarding data availability: The raw data are available in the Supplemental Files.

## References

[ref-1] Cheng M, Zhu QL, Liu L, An SS (2013). Effects of vegetation on soil aggregate stability and organic carbon sequestration in the Ningxia Loess Hilly Region of northwest China. Acta Ecologica Sinica.

[ref-61] Gai X, Zhang XP, Bian FY, Yang CB, Zhong ZK (2020). Research progress in heavy metal remediation mechanism by microorganisms in forest soil. World Forestry Research.

[ref-2] Golhar AR, Choudhari NK, Patil AK (2021). Prediction of aluminium content in a metal using SPSS based linear regression analysis. Journal of Physics: Conference Series.

[ref-3] Guo JK, Zhao JJ, Li YF, Liu X, Liu T, Niu YH, Li X (2022). Research progress on remediation technology for heavy metal-contaminated soil in mines. Journal of Agricultural Resources and Environment.

[ref-4] Han ZX, Wang LS, Guo JQ, Li XY, Ma YN, Liu XY (2012). Heavy metal forms in the process of soil remediation. Acta Petrologica et Mineralogica.

[ref-5] Han YY, Wang HJ, Zhang GM, Zhang SQ, Liu XC, Ling Liu (2022). Distribution, ecological risk assessment and source identification of pollutants in soils of different land-use types in degraded wetlands. PeerJ.

[ref-6] Han RY, Xu ZF (2022). Spatial distribution and ecological risk assessment of heavy metals in karst soils from the Yinjiang County, Southwest China. PeerJ.

[ref-7] He XJ, Wang L, Ke B, Yue YM, Wang KL, Cao JH, Xiong KN (2019). Progress on ecological conservation and restoration for China Karst. Acta Ecologica Sinica.

[ref-8] He GD, Zhang ZM, Wu XL, Cui MY, Zhang JC, Huang XF (2020). Adsorption of heavy metals on soil collected from lixisol of typical karst areas in the presence of CaCO3 and soil clay and their competition behavior. Sustainability.

[ref-9] Jitendra M, Rachna S, Naveen KA (2017). Alleviation of heavy metal stress in plants and remediation of soil by rhizosphere microorganisms. Frontiers in Microbiology.

[ref-10] Konyshev AA, Sidkina ES, Cherkasova EV, Mironenko MV, Gridasov AG, Zhilkina AV, Bugaev IA (2020). Migration forms of heavy metals and chemical composition of surface waters in the arsenic shaft area (Pitkäranta Ore District, South Karelia). Geochemistry International.

[ref-11] Li JF (2020). Research progress of heavy metal pollution hyperaccumulator in mining area at home. Conservation and Utilization of Mineral Resources.

[ref-12] Li JM, Jin ZX, Gu QP (2011). Effect of plant species on the function and structure of the bacterial community in the rhizosphere of lead-zinc mine tailings in Zhejiang, China. Canadian Journal of Microbiology.

[ref-13] Li YT, Thierry B, Dai J, Benedetti MF (2009). Ion activity and distribution of heavy metals in acid mine drainage polluted subtropical soils. Environmental Pollution.

[ref-14] Li TQ, Yang XE, Jin XF, Hu QH (2005). Root responses and metal accumulation in two contrasting ecotypes of Sedum alfredii Hance under lead and zinc toxic stress. Journal of Environmental Science and Health—Part A Toxic/Hazardous Substances and Environmental Engineering.

[ref-15] Li WJ, Zhu TB, Yang H, Zhang CL, Zou X (2022). Distribution characteristics and risk assessment of heavy metals in soils of the typical karst and non-karst areas. Land.

[ref-16] Lin H, Tang YL, Dong YB, Wei ZS, Liu CJ (2022). Characterization of heavy metal migration, the microbial community, and potential bioremediating genera in a waste-rock pile field of the largest copper mine in Asia. Journal of Cleaner Production.

[ref-17] Liu YL, Liu SL, Wu M, Tian XL, Liu SY (2022). The effect of land use/land cover change on the concentration of se and heavy metals in soils from an area return cropland to forest, Southwest China. Environmental Science.

[ref-18] Ma YL, He JL, Ma CF, Luo J, Li H, Liu TX, Polle A, Peng CH, Luo ZB (2014). Ectomycorrhizas with Paxillus involutus enhance cadmium uptake and tolerance inPopulus × canescens. Plant, Cell and Environment.

[ref-19] Ma Y, Prasad MNV, Rajkumar M, Freitas H (2010). Plant growth promoting rhizobacteria and endophytes accelerate phytoremediation of metalliferous soils. Biotechnology Advances.

[ref-62] National Forestry and Grassland Administration of China (1987). Determination of Microaggregate Composition of Forest Soil (GB 7846-1987).

[ref-20] Qi YT, Wei XD, Zhao MJ, Pan WS, Jiang C, Wu JB, Li WC (2022). Heavy metal pollution characteristics and potential ecological risk assessment of soils around three typical antimony mining areas and watersheds in China. Frontiers in Environmental Science.

[ref-21] Sizmur T, Hodson ME (2009). Do earthworms impact metal mobility and availability in soil?—a review. Environmental Pollution.

[ref-22] Su CY, Wang JW, Chen ZW, Meng J, Yin GC, Zhou YQ, Wang TY (2022). Sources and health risks of heavy metals in soils and vegetables from intensive human intervention areas in South China. The Science of the Total Environment.

[ref-23] Sun J, Wang XF, Liu CH, Zhao YS (2009). Correlation and change between soil nutrient and heavy metal in graphite tailings wasteland during vegetation restoration. Journal of Soil and Water Conservation.

[ref-24] Sun CL, Zhu SX, Zhao B, Li WJ, Gao XY, Wang XD (2020). Effect of land use conversion on surface soil heavy metal contamination in a typical Karst Plateau Lakeshore Wetland of Southwest China. International Journal of Environmental Research and Public Health.

[ref-25] Tang SQ, Liu XJ, Yang K, Guo F, Yang Z, Ma HH, Li K (2021). Migration, transformation characteristics, and ecological risk evaluation of heavy metal fractions in cultivated soil profiles in a typical carbonate-covered area. Environmental Science.

[ref-26] Walkley A, Black IA (1934). An examination of the Degtjareff method for determining soil organic matter, and a proposed modification of the chromic acid titration method. Soil Science.

[ref-27] Wang Y, Feng FY, Ge J, Li Y, Yu XY (2022a). Effects and mechanisms of plant root exudates on soil remediation. Acta Ecologica Sinica.

[ref-28] Wang Y, Huang XF, Hu JW, Xiong KN, Duan SM (2013). Accumulation of heavy metals by wetland plants with different root systems in a Karst Mountainous Area. Advanced Materials Research.

[ref-29] Wang QL, Song YT, Wang CW, Peng M, Han W, Zhou YL (2021). Characteristics and genesis of soil element background Baoshan-Lincang Area in Western Yunnan Province. Journal of Kunming University of Science and Technology (Natural Sciences).

[ref-30] Wang YM, Tan SN, Mohd Yusof ML, Ghosh SH, Lam YM (2022b). Assessment of heavy metal and metalloid levels and screening potential of tropical plant species for phytoremediation in Singapore. Environmental Pollution.

[ref-31] Wei LL, Wang K, Daniel R (2016). Transformation and speciation of typical heavy metals in soil aquifer treatment system during long time recharging with secondary effluent: depth distribution and combination. Chemosphere.

[ref-32] Wu YJ, Tian X, Zhang MY, Wang S (2022a). Influence of vegetation restoration on carbon in the lakeside zone of Karst Wetland in Guizhou Plateau. Acta Ecologica Simica.

[ref-33] Wu YJ, Tian X, Zhang MY, Wang RZ, Shuo W (2022b). A case study of initial vegetation restoration affecting the occurrence characteristics of phosphorus in Karst geomorphology in Southwest China. Sustainability.

[ref-34] Wu H, Zhang JL, Fan YW, Yu LF, Yan LB, Yuan CJ (2013). Numerical classification and ordination of forest communities in Caohai basin. Journal of Nanjing Forestry University (Natural Sciences Edition).

[ref-35] Xie HX, Ren ZY, Li R (2007). Economic value of vegetation carbon fixation and oxygen release in Loess Plateau of North Shaanxi Province under land-use and land-cover change. Chinese Journal of Ecology.

[ref-36] Xie LW, Zhong J, Cao FX, Cao FX, Li JJ, Wu LC (2015). Evaluation of soil fertility in the succession of karst rocky desertification using principal component analysis. Solid Earth.

[ref-37] Xu T, Xu Y, Jiang B, Zhang L, Song WB, Zhou DM (2015). Evaluation of the ecosystem services in Caohai Wetland, Guizhou Province. Acta Ecologica Sinica.

[ref-38] Xu L, Zhou J, Zhang WH, Cui BH, Liu HL, Liu CH, Liang JN, Zhou J (2017). Effects of vegetation restoration on soil organic matter and aggregate characteristics of heavy metal contaminated soils. Research of Soil and Water Conservation.

[ref-39] Yang QQ, Li ZY, Lu XN, Duan QQ, Huang L, Bi J (2018). A review of soil heavy metal pollution from industrial and agricultural regions in China: pollution and risk assessment. Science of the Total Environment.

[ref-40] Yang LY, Wu P, Yang WT (2022). Characteristics, health risk assessment, and transfer model of heavy metals in the soil—food chain in cultivated land in karst. Foods.

[ref-41] Yang L, Xin JP, Tian RN (2022). Research Progress in the mitigative effects of rhizosphere microorganisms on heavy metal Stress in plants and their mechanisms. Biotechnology Bulletin.

[ref-42] Yao CB, Zhou MZ, Xiong KN, Zhang D, Yang H, Zhang XR, Yang LS (2021). Contents of heavy metals in soils and crops in the demonstration area of karst rocky desertification control of the Karst Plateau-Gorge. China Environmental Science.

[ref-43] Zeng FR, Chen S, Miao Y, Wu F, Zhang G (2008). Changes of organic acid exudation and rhizosphere pH in rice plants under chromium stress. Environmental Pollution.

[ref-44] Zeng J, Han GL, Wu QX, Tang Y (2019). Geochemical characteristics of dissolved heavy metals in Zhujiang River, Southwest China: spatial-temporal distribution, source, export flux estimation, and a water quality assessment. PeerJ.

[ref-45] Zhang R, Chen T, Zhang Y, Hou YH, Chang QR (2020a). Health risk assessment of heavy metals in agricultural soils and identification of main influencing factors in a typical industrial park in northwest China. Chemosphere.

[ref-46] Zhang Q, Han GL (2022). Speciation characteristics and risk assessment of soil heavy metals from Puding Karst critical zone, Guizhou Province. Environmental Science.

[ref-47] Zhang YL, Li J, Leng ZR, Du DL (2020b). The influence of root exudate flavonoids on sulfur species distribution in mangrove sediments polluted with cadmium. Wetlands.

[ref-48] Zhang JR, Li HZ, Zhou YZ, Dou L, Cai LM (2018). Bioavailability and soil-to-crop transfer of heavy metals in farmland soils: a case study in the Pearl River Delta, South China. Environmental Pollution.

[ref-49] Zhang QY, Liu HY, Liu F, Ju XH, Dinis F, Yu EJ, Yu Z (2022). Source identification and superposition effect of heavy metals (HMs) in agricultural soils at a high geological background area of karst: a case study in a typical watershed. International Journal of Environmental Research and Public Health.

[ref-50] Zhang JC, Mu GT, Zhang ZM, Huang XF, Fang H (2021). Speciation variation and bio-activation of soil heavy metals (Cd and Cr) in rice-rape rotation lands in Karst Regions. International Journal of Environmental Research and Public Health.

[ref-51] Zhang JL, Wu H, Yu LF, Yan LB, Yuan CJ, Cai GJ (2013). Analysis of α and β diversity of typical karst forest communities in the Caohai Wetland Basin. Forest Science and Technology.

[ref-52] Zhang JC, Zeng XP, Zhang ZM, Wen XM, Zhang QH, Lin CH (2019). Characteristics and evalution of speciation of heavy metal in forest of Karst. Research of Soil and Water Conservation.

[ref-53] Zhang XM, Zhang XY, Zhong TY, Jaing H (2014). Spatial distribution and accumulation of heavy metal in arable land soil of China. Environmental Science.

[ref-54] Zhao FJ, Ma YB, Zhu YG, Zhong T, Mcgrath SP (2015). Soil contamination in China: current status and mitigation strategies. Environmental Science and Technology.

[ref-55] Zhong TY, Xue DW, Zhao LM, Zhang XY (2018). Concentration of heavy metals in vegetables and potential health risk assessment in China. Environmental Geochemistry and Health.

[ref-56] Zhou PF, Zhang SW, Luo M, Wei HB, Song Q, Fang B, Zhuang HJ, Chen HY (2022). Characteristics of plant diversity and heavy metal enrichment and migration under different ecological restoration modes in abandoned mining areas. Environmental Science.

[ref-57] Zhu GX, Xiao HY, Guo QJ (2018). Heavy metal contents and enrichment characteristics of dominant plants in wasteland of the downstream of a lead-zinc mining area in Guangxi, Southwest China. Ecotoxicology and Environmental Safety.

